# Impact of Clinical Decision Support on Radiography for Acute Ankle Injuries: A Randomized Trial

**DOI:** 10.5811/westjem.2017.1.33053

**Published:** 2017-03-07

**Authors:** Shahein Tajmir, Ali S. Raja, Ivan K. Ip, James Andruchow, Patricia Silveira, Stacy Smith, Ramin Khorasani

**Affiliations:** * Brigham and Women’s Hospital, Center for Evidence-Based Imaging, Boston, Massachusetts; † Brigham and Women’s Hospital, Department of Radiology, Boston, Massachusetts; ‡ Brigham and Women’s Hospital, Department of Emergency Medicine, Boston, Massachusetts; § Brigham and Women’s Hospital, Department of Medicine, Boston, Massachusetts; ¶ Massachusetts General Hospital, Department of Emergency Medicine, Boston, Massachusetts; || Massachusetts General Hospital, Department of Radiology, Boston, Massachusetts; # Harvard Medical School, Boston, Massachusetts

## Abstract

**Introduction:**

While only 15–20% of patients with foot and ankle injuries presenting to urgent care centers have clinically significant fractures, most undergo radiography. We examined the impact of electronic point-of-care clinical decision support (CDS) on adherence to the Ottawa Ankle Rules (OAR), as well as use and yield of foot and ankle radiographs in patients with acute ankle injury.

**Methods:**

We obtained institutional review board approval for this randomized controlled study performed April 18, 2012—December 15, 2013. All ordering providers credentialed at an urgent care affiliated with a quaternary care academic hospital were randomized to either receive or not receive CDS, based on the OAR and integrated into the physician order-entry system, with feedback at the time of imaging order. If the patient met OAR low-risk criteria, providers were advised against imaging and could either cancel the order or ignore the alert. We identified patients with foot and ankle complaints via ICD-9 billing codes and electronic health records and radiology reports reviewed for those who were eligible. Chi-square was used to compare adherence to the OAR (primary outcome), radiography utilization rate and radiography yield of foot and ankle imaging (secondary outcomes) between the intervention and control groups.

**Results:**

Of 14,642 patients seen at urgent care during the study period, 613 (4.2%, representing 632 visits) presented with acute ankle injury and were eligible for application of the OAR; 374 (59.2%) of these were seen by control-group providers. In the intervention group, CDS adherence was higher for both ankle (239/258=92.6% vs. 231/374=61.8%, p=0.02) and foot radiography (209/258=81.0% vs. 238/374=63.6%; p<0.01). However, ankle radiography use was higher in the intervention group (166/258=64.3% vs. 183/374=48.9%; p<0.01), while foot radiography use (141/258=54.6% vs. 202/374=54.0%; p=0.95) was not. Radiography yield was also higher in the intervention group (26/307=8.5% vs. 18/385=4.7%; p=0.04).

**Conclusion:**

Clinical decision support, previously demonstrated to improve guideline adherence for high-cost imaging, can also improve guideline adherence for radiography – as demonstrated by increased OAR adherence and increased imaging yield.

## INTRODUCTION

Patients with foot and ankle pain often present to emergency departments (ED) and urgent care centers, accounting for nearly 2.8 million visits in 2010 (7.6% of all injury visits).^1^ Despite this frequency, clinically significant fractures are only found in 15–20% of cases.^2^ Validated, high quality, evidence-based guidelines for imaging patients with suspected ankle fracture (the Ottawa Ankle Rules [OAR]),^3^,^4^,^2^ have been available for almost 20 years. However, their widespread adoption into practice has been suboptimal. In 2001, 96% of United States (U.S.) physicians reported familiarity with the rules; however, only 31% reported using them “always” or “most of the time.”^5^ In the same study, Canadian physicians reported using the rules 89% of the time. However, despite this, an analysis in Ontario showed that ED foot and ankle radiography still increased 1% annually from 2001–2007.^6^

The federal Health Information Technology and Economic Health (HITECH) Act of 2009 aims to improve quality of healthcare and reduce waste through meaningful use of health IT, including a major focus on clinical decision support (CDS).^7^,^8^ Imaging CDS and CDS-enabled interventions have been reported to improve adherence to evidence^9^,^10^,^11^ and to reduce unnecessary imaging and increase its yield.^12^–^16^,^17^,^18^ Imaging CDS is most effective when based on high-quality evidence and embedded in provider workflow.^19^ However, most prior reports have focused on impact of CDS on “high cost” imaging (e.g., computed tomography, magnetic resonance imaging) with sparse use of a randomized controlled study design.^20^ Despite the emphasis on high-cost imaging, low-cost imaging examinations are the most common diagnostic imaging examinations performed in the U.S. and may be overused^21^,^22^, expose the patient to unnecessary ionizing radiation,^23^,^24^, may result in longer length of stay in the ED,^25^,^26^ and result in incidental or ambiguous findings that lead to additional high-cost imaging.^27^ However, it remains unclear whether CDS will have a similar impact in low-cost as in high-cost imaging.

Thus, the purpose of this study was to assess the impact of CDS on radiography for acute ankle and foot injuries. More specifically, we evaluated the impact of a CDS tool on physician-documented adherence to the OAR in the evaluation of acute ankle injury in the urgent care setting. We chose the urgent care setting, as such centers are typically designed to handle relatively low acuity injuries (e.g., acute ankle and foot injuries). We hypothesized that such CDS, integrated into provider workflow, would increase adherence to the OAR.

## METHODS

### Study Setting and Subjects

We obtained institutional review board approval for this Health Insurance Portability and Accountability Act-compliant randomized controlled study, performed between April 18, 2012, and December 15, 2013, at an urgent care center affiliated with a quaternary care, academic hospital. All providers (medical doctors and physician assistants) credentialed at the urgent care center, stratified by title, were randomized to either receive the CDS intervention at the time of ordering a foot or ankle radiograph (intervention group) or not (control group). Providers who began working at the urgent care center after the randomization period were excluded from the study.

**Population Health Research Capsule**

What do we already know about this issue?

Clinical decision support (CDS) has been effective for improving the appropriateness of high-cost imaging, but its effect on low-cost imaging remains unclear.

What was the research question?

What was the impact of randomized CDS on adherence to the Ottawa Ankle Rules (OAR)?

What was the major finding of the study?

While adherence to the OAR increased with CDS, use of ankle radiographs was also higher in the CDS group.

How does this improve population health?

Evidence-based CDS can be successfully implemented for both low- and high-cost imaging in the ED.

### Data Collection

Although providers were prospectively randomized, we collected data retrospectively. Data were captured concurrently with patient care, including in the CDS system for providers randomized to receive it. Therefore, we waited until all study data accrued and then collected it. Using International Classification of Diseases, Ninth Revision (ICD-9) codes, we queried the billing database for all unique patients presenting to the urgent care center during the study period with a discharge diagnosis code for a foot or ankle complaint (719.47, 824.x, 825.x, 826.x, 829.x, 837.0, 838.0, 845.x, 924.2x, and 928.2x; see Supplemental eTable 1). A subsequent review of each patient’s electronic health record (EHR) was performed using an explicit chart review data collection form. Data collected included patient age, chief complaint, mechanism of injury, presence of any of the exclusion criteria from the OAR (injury greater than 10 days prior to presentation, altered level of consciousness, multiple trauma, pregnancy, penetrating or open wounds, patients with isolated superficial skin complaints, and patients < 18 years old), tenderness over the malleoli or midfoot, and radiography utilization (eTable 2). We reviewed radiology reports to determine whether a fracture was noted and, if so, what type. Patients with chief complaints not pertaining to the foot or ankle were excluded. After chart review, patients with one or more OAR exclusion criterion were removed from the study cohort. We included only the first patient visit for each trauma episode; patient visits for re-assessment of the same ankle or foot injury were excluded.

### Intervention

**Figure 1A–E:**
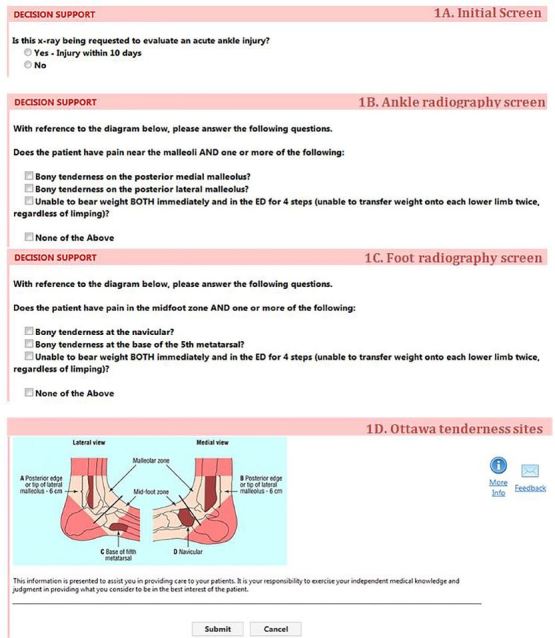

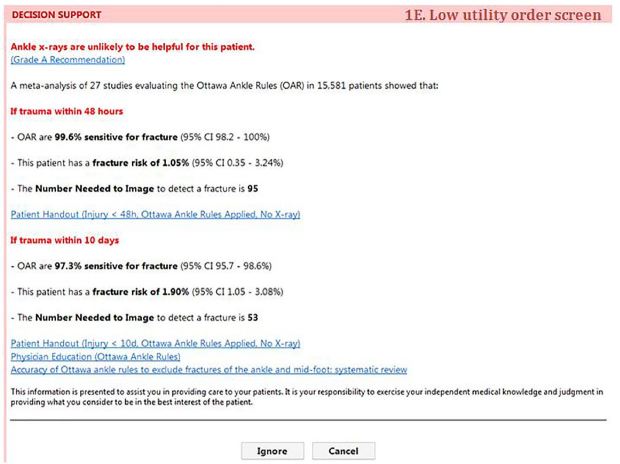
Clinical decision support screens for the Ottawa ankle rule integrated in the computerized physician order-entry system. *ED,* emergency department

Providers were clustered into two subsets based on their titles (physician [MD] or physician assistant [PA]). In April 2012, providers in each subset were randomly assigned to the intervention or control groups using a random number generator function. Intervention group urgent care providers were exposed to CDS based on the OAR integrated into the computerized physician order entry (CPOE) system (Percipio; Medicalis, San Francisco CA). The CDS intervention consisted of four successive screens to capture data to determine the utility of the study according to the OAR (Figure 1A–D), and one educational screen (Figure 1E). If the utility of the study was low, the provider was exposed to the educational screen informing him/her of such, and s/he could either cancel or proceed with the imaging order.

To determine whether any differences observed in adherence were simply a result of “gaming” the system, which we defined as inaccurate data entry into the CPOE system to avoid potentially onerous CDS interactions, one investigator (IKI) performed manual chart reviews of 158 randomly sampled charts in the CDS group, based on power calculation with alpha of 0.05, power of 0.8, and confidence interval of 15%. We calculated the concordance and discordance rates between data in the physician note and data entered into the order entry screen (electronic orders consisting of the data entered in CPOE and CDS). Visits are concordant when the data in the visit note and the CPOE and/or CDS system matched (based on adherence to the evidence-based guideline). They are discordant when data are conflicting. If data entered in the CPOE and/or CDS system did not have corresponding entry in the physician note, we considered the visit to have incomplete documentation in the physician note rather than as discordant.

### Outcome Measures

The primary outcome measure was adherence to the OAR, defined as the number of eligible patients with acute ankle and foot injuries whose workup was consistent with the OAR, whether or not they were imaged. We calculated the adherence rate for each group.

Secondary outcome measures were radiography utilization and fracture rates (radiography yield) among patients with acute ankle injuries. Ankle fracture, foot fracture, and clinically significant fracture rates were calculated. We defined clinically significant fractures as those with fracture displacement greater than 3 mm^2^–^4^. Radiography yield was calculated as the number of foot (or ankle) radiographs that detected a clinically significant fracture divided by the total number of foot (or ankle) radiographs in each group. We calculated radiography utilization rate as the number of foot (or ankle) radiographs performed divided by the total number of patient visits for acute ankle injury in each group.

Power calculation based on the rate of adherence to OAR estimated that a sample size of 334 patient visits (167 in each arm) was needed to detect a relative difference of 15% between the intervention and control groups (baseline adherence of 0.5^10^,^14^, alpha = .05, power = 0.8).

### Statistical Analysis

We descriptively analyzed provider demographics. Chi-square was used to compare adherence to the OAR, radiography utilization rate and radiography yield of foot and ankle imaging between the intervention and control groups. We considered a two-tailed p-value of <0.05 statistically significant. All analyses were conducted using JMP Pro 11.0 (SAS Institute, Cary, NC).

## RESULTS

### Provider and Patient Characteristics

A total of 66 providers were randomized to either the intervention (n=32) or control (n=34) groups; 26 of them (10 intervention, 16 control) saw eligible patients during the study period (Table 1). There were 22,982 total visits (14,642 unique patients) to the urgent care center during the 20-month study period. Of these, 988 patients were identified by the ICD-9 codes pertaining to acute ankle and foot injuries, representing 6.7% of all patients seen. We excluded 356 visits (1.5%) identified by ICD-9 codes as foot and ankle related: 177 visits were not associated with an ankle injury, 7 were multi-trauma, 26 were isolated injuries to the skin, 13 were referred with radiographs, 81 were injuries that happened more than 10 days previously, 44 were reassessment of the same injury, and 8 were associated with pregnant patients. After applying exclusion criteria, 613 patients (4.2% of all patients) representing 632 patient visits (2.7% of all patient visits) were clinically eligible for application of the OAR, of which 258 patient visits (40.8% of eligible visits) were seen by a provider in the intervention group.

Discordance between EHR and order requisition was 0% in the control and 1.3% in the intervention group while concordance was 48% and 10.7%, respectively. In the remaining cases, EHR documentation of discrete clinical information entered in CPOE was incomplete to unambiguously assess adherence to OAR.

### Adherence to Evidence-based Guidelines

Rate of adherence to the OAR was higher for ankle radiography (92.6% vs. 61.8%, p=0.015) and foot radiography (81.0% vs. 63.6%, p<0.001) in the intervention group as compared to the control group (Table 2).

### Radiography Use

Ankle radiography use was higher in the intervention group (64.3% vs. 48.9%, p<0.001), but foot radiography use (54.6% vs. 54.0%, p=0.950) was not significantly different (Table 3). Ankle and foot radiography was performed in 25.2% of patient visits in the intervention group compared to 15.8% in the controls (p<0.01. Only 6.6% of patient visits in the intervention group had no radiography, compared to 12.6% in the control group (p=0.0136)

### Fracture Prevalence

Prevalence of clinically significant fractures in the study was 8.7% (44/632), 2.1-fold higher in the intervention group (10.1% vs. 4.8%, p<0.02). Significant ankle fractures were 1.9-fold higher in the intervention group (4.7% vs. 2.4%, p=0.122) while significant foot fractures were 2.3-fold higher in the intervention group (5.4% vs. 2.4%, p<0.05).

Prevalence of all fractures in the study was 13.3% (84/632), twofold higher in the intervention group (19.0% vs. 9.4%, p<0.01). A total of 48 ankle fractures were identified in the cohort (7.59%). The prevalence rate of all ankle fractures was 1.9-fold higher in the intervention group (10.5% vs. 5.6%, p<0.02). A total of 36 foot fractures (5.70%) were identified in the cohort. The prevalence rate of all foot fractures was 2.3-fold higher in the intervention group (8.5% vs. 3.7%, p<0.01; Table 4).

### Radiography Yield

For clinically significant fractures, the radiography yield was 1.8-fold higher in the intervention group (overall 26/307=8.5% vs. 18/385=4.7%, p=0.0421). Foot radiography yield was 2.2-fold higher (14/141=9.9% vs. 9/202=4.5%, p=0.0461). Ankle radiography yield was 1.5-fold higher but did not reach significance (12/166=7.2% vs. 9/183=4.9%, p=0.354).

For all fractures, the radiography yield was 1.8-fold higher in the intervention group (overall 49/307=16.0% vs. 35/385=9.1%, p=0.0060). Foot radiography yield was 2.3-fold higher (22/141=15.6% vs. 14/202=6.9%, p=0.0099). Ankle radiography yield was 1.4-fold higher but did not reach significance (27/166=16.2% vs. 21/183=11.5%, p=0.195; Table 4).

## DISCUSSION

Foot and ankle radiography represent low-cost, high-volume tests that – when used inappropriately – create waste, unnecessary radiation exposure, and likely increased lengths of stay in the ED/urgent care center.^4^ Similar to concerns regarding inappropriate use of high-cost imaging, if radiography imaging results are ambiguous or if incidental findings are discovered, potentially unnecessary downstream diagnostic and therapeutic procedures (with their associated costs and risks) may result.

We found that the implementation of a CDS tool at an urgent care center resulted in a significant increase in documented adherence to OAR, improving adherence to 93% for ankle and 81% for foot radiography for acute ankle injuries. Although we did not quantitatively compare the effort required to implement and sustain OAR deployment, associated data capture, and unambiguous calculation of adherence to OAR when using CDS compared to paper-based interventions, the relative ease of performing our experiment may encourage implementation of other decision rules using CDS infrastructure. Moreover, prior reports highlight the need for chart review when using paper forms to complete data extraction from the patient’s chart as nearly 23% of data capture forms were incomplete.^4^,^28^ When using CDS, providers were required to complete entry of required data elements to place orders for imaging. Quality improvement strategies using CDS may thus provide near real-time measure of provider’s adoption of evidence without the need for time-intensive retrospective chart review.

We also found that implementation of CDS reduced unnecessary foot radiography. Although the prevalence of clinically significant foot fractures was 2.3-fold higher in the CDS group, foot radiography use was similar to the control group, resulting in a higher foot radiography yield in the CDS group.

Our findings suggest overutilization of radiography even post CDS. Prior studies have reported approximately 16% prevalence of significant fractures, and 4% prevalence of avulsion fractures in an ED cohort of patients with acute ankle injuries^4^,^28^. The intervention group in our urgent care had a 10% prevalence of significant fractures (with a nearly equal proportion of avulsion injuries), reflecting the diagnosis of less severe injuries compared to the ED. However, despite these less severe injuries, patients in our CDS group were imaged more frequently than prior reports. Stiell et al. reported that 20% of patients were spared imaging, with 10% having both ankle and foot imaging after implementation of OAR. Conversely, in our CDS cohort, only 6.6% of patients were spared imaging and 25% had both ankle and foot imaging (see eTable 4 for comparisons to previously published data). In our intervention group, 241 patients were imaged to identify 12 clinically significant ankle and another 14 clinically significant foot fractures. The overuse of imaging was even more dramatic in the control group: 327 patients were imaged to diagnose nine clinically significant ankle and another nine clinically significant foot fractures.

Our results suggest that despite the existence of a well-known, validated decision rule, use of radiography for the evaluation of acute ankle trauma in the urgent care setting is suboptimal. Moreover, we found that although implementation of CDS based on OAR resulted in modest improvement in use of radiography in these patients, radiography use was not optimized. This overuse of imaging may be due to a number of factors. Patients’ preferences for imaging may have been a contributing factor when evidence-based guidelines were not followed; while patients are becoming aware of the risks of high cost high-radiation imaging, extremity radiographs carry a much less negative connotation. In addition, the OAR may have been suboptimally applied. Future studies would be needed to assess whether additional teaching, to both patients and providers, on use of OAR might reduce unnecessary utilization of radiography in patients with acute ankle injuries in urgent care centers.

The lower concordance between CDS-documented clinical attributes and the physician note found in the CDS group is expected. CDS required explicit documentation of relevant discrete clinical attributes, a capability absent in narrative documentation in the physician notes. This limitation of physician notes highlights the shortcomings of some current strategies for data collection (which rely on automated data extraction strategies from EHRs) as information may not be well documented and will thus often not be discoverable.

## LIMITATIONS

There were a number of limitations to our study. We were unable to assess impact of OAR embedded in CDS on use and yield of radiography for evaluation of ankle fractures. The prevalence of significant fractures of ankle and foot differed significantly (near twofold) between control and intervention groups. Thus, our observed higher imaging yield of significant fractures, and higher use of radiography in the intervention group may simply reflect the higher prevalence of fractures in the intervention group. This in turn suggests that our randomization might not have been effective, with the intervention group consisting of providers evaluating patients with more significant fractures. An alternative explanation would be that a substantial number of significant fractures were missed in the control group, a very unlikely scenario, as 90% of the patients enrolled in the study were imaged, and we found no missed fractures re-presenting to the urgent care center. Secondly, we randomized on an intent-to-treat basis. As all clinicians were randomized based on being credentialed to practice at the urgent care, not every physician and PA may have worked there during the study period. Thirdly, our data was all obtained from a single site and thus may not be generalizable. Finally, we did not train our providers in interpreting OAR, which may have contributed to overuse of radiography.

## CONCLUSION

We found that implementation of the Ottawa Ankle Rules embedded in clinical decision support significantly improved documented adherence to the OAR, and modestly improved use of foot radiography. The relative ease of implementation, data capture, and unambiguous measurement of provider adherence to OAR, without the need for time-consuming chart review, suggests CDS can efficiently deliver complex imaging-related decision rules embedded in provider workflow. Despite more than 20 years of experience with OAR,^4^,^28^, we found radiography likely remains overused in patients with acute ankle injury in urgent care centers. Future studies would be needed to assess whether additional training about the Ottawa Ankle Rules for providers and patients, or more stringent CDS-enabled interventions, can help reduce unnecessary radiography in these patients.

## Supplementary Information



## Figures and Tables

**Table 1:** Provider characteristics by group and total in study of efficacy of clinical decision support, based on the Ottawa Ankle Rules, to curb unnecessary foot and ankle radiography.

	CDS		Control		Overall	
			
	N	%	N	%	N	%
Initial randomization						
MD	23		24		47	
Physician assistant	9		10		19	
Total randomized	32		34		66	100.0%
Providers who saw patients*						
MD	4		12		16	
Physician assistant	6		4		10	
Total at end of study	10	38.5%	16	61.5%	26	100.0%
Patient distribution						
MD	162		265		427	
Physician assistant	96		109		205	
Total patients seen	258	40.8%	374	59.2%	632	100.0%
Provider characteristics						
Average years of experience	13		16			
MD	19		18.5			
PA	10		9.3			
Gender (% male)		50%		56%		
MD		75%		75%		
PA		43%		0%		

*CDS,* clinical decision support.

* Providers randomized to each group who actually evaluated enrolled patients during the study.

**Table 2:** Adherence to Ottawa Ankle Rules (OAR) and radiography use by group.

Adherence	CDS intervention group			Control group			
			
	Workups consistent with OAR	N	Adherence	Workups consistent with OAR	N	Adherence	p-value
Ankle	239	258	92.6%	231	374	61.8%	0.0155*
Foot	209	258	81.0%	238	374	63.6%	0.0001*
Use	CDS intervention group			Control group			
	No. exams performed	Patients seen	Use	No. exams performed	Patients seen	Use	p-value
Ankle only	101	258	39.1%	124	374	33.2%	0.1375
Foot only	76	258	29.5%	143	374	38.2%	0.0194
Ankle and Foot	65	258	25.2%	59	374	15.8%	0.0039
No radiography	17	258	6.6%	47	374	12.6%	0.0134
Total Ankle	166	258	64.3%	183	374	48.9%	0.0002*
Total Foot	141	258	54.6%	202	374	54.0%	0.95

*CDS,* clinical decision support; OAR, Ottawa Ankle Rules.

* Values are statistically significant.

**Table 3:** Radiography combinations by group.

	Clinical decision support intervention group			Control group			
			
	No. exams performed	Patients seen	%	No. of exams performed	Patients seen	%	p-value
Ankle only	101	258	39.1%	124	374	33.2%	0.1375
Foot only	76	258	29.5%	143	374	38.2%	0.0194
Ankle and foot	65	258	25.2%	59	374	15.8%	0.0039
None	17	258	6.6%	47	374	12.6%	0.0134

**Table 4:** Radiography yield and fractures per patient visit of ankle and foot radiography by group.

	CDS intervention group					Control						
				
	Fractures	Total exams	Yield	# Patient visits	Fractures per Patient visit	Fractures	Total exams	Yield	# Patient visits	Fractures per patient visit	Yield p-values	Patient visit p-values
Clinically significant fractures	26	307	8.7%	258	10.1%	18	385	4.7%	374	4.8%	0.0421	0.0165
Ankle	12	166	7.2%	258	4.7%	9	183	4.9%	374	2.4%	0.364	0.122
Foot	14	141	9.9%	258	5.4%	9	202	4.5%	374	2.4%	0.0461	0.0463
Avulsion fractures	23	307	7.5%	258	8.9%	17	385	4.4%	374	4.5%	0.0849	0.0266
Ankle	15	166	9.0%	258	5.8%	12	183	6.6%	374	3.2%	0.389	0.111
Foot	8	141	5.7%	258	3.1%	5	202	2.5%	374	1.3%	0.127	0.125
All fractures	49	307	16.0%	258	19.0%	35	385	9.1%	374	9.4%	0.0060	0.0004
Ankle	27	166	16.2%	258	10.5%	21	183	11.5%	374	5.6%	0.195	0.0237
Foot	22	141	15.6%	258	8.5%	14	202	6.9%	374	3.7%	0.0099	0.0108

*CDS,* clinical decision support.
