# Supplementary material for: Impact of Clinical Decision Support on Radiography for Acute Ankle Injuries: A Randomized Trial
Source: West J Emerg Med. 2017 Mar 7;18(3):487–95. doi: 10.5811/westjem.2017.1.33053 (PMC5391900; doi:10.5811/westjem.2017.1.33053)
Supplement: Supplementary file 1 [file wjem-18-487-s001.pdf]

Online Supplements

**eTable 1: ICD9 Codes used to identify patients with acute foot or ankle complaints.**	
719.47	Pain in Joint, Ankle or Foot
824.x	Fracture of Ankle
825.x	Fracture Tarsal/Metatarsal
826.x	Fracture of Phalanges
829	Fracture Unspecified
837	Dislocation of Ankle
838	Dislocation of Foot
845.x	Sprain of Ankle or Foot
924.2x	Contusion of Ankle or Foot
928.2x	Crush Injury of Ankle or Foot

**eTable 2: Information abstracted from chart review**	
Medical Record Number	Inability to bear weight for 4 steps
Date of Service	TTP posterior medial malleolus
Attending MD	TTP posterior lateral malleolus
PA (if applicable)	TTP navicular
Age	TTP base of 5th metatarsal
Chief Complaint	Ankle X-ray?
Mechanism of Injury	Ankle Fracture?
Injury within 10 days	Foot X-ray?
Exclusions (Polytrauma, Penetrating Trauma, Paraplegia, Pregnancy, Reassessment)	Foot fracture?

eTable 3: Fracture locations for all ankle and foot injury patients seen at the urgent care center during the 20-month study period

	Control		CDS		Overall	
	N	%	N	%	N	%
Total patients	374	59.18%	258	40.82%	632	100.00%
**Significant Fractures**	18	4.81%	26	10.08%	44	6.96%
**Malleolar Region**	9	2.41%	12	4.65%	21	
Lateral malleolus	4		11		15	
Medial malleolus	2		0		2	
Posterior malleolus	0		0		0	
Talus	0		0		0	
Bimalleolar	3		0		3	
Trimalleolar	0		1		1	
**Midfoot**	9	2.41%	14	5.43%	23	3.64%
Base 5th metatarsal	8		10		18	
Navicular	1		4		5	
Anterior process calcaneus	0		0		0	
Cuboid	0		0		0	
Cuneiforms	0		0		0	
**Avulsion Fractures**	17	4.55%	23	8.91%	40	6.33%
Lateral malleolus	6		4		9	
Lateral malleolus SHI	0		2		2	
Lateral malleolus SHII	1		2		3	
Medial malleolus	0		2		2	
Posterior malleolus	1		0		1	
Posterior malleolus SHII	0		1		1	
Talus	4		4		8	
Base 5th metatarsal	1		1		2	
Navicular	3		5		7	
Anterior process calcaneus	1		0		1	
Cuboid	0		1		1	
Cuneiforms	0		1		1	

eTable 4. Comparisons to previously published data

	Pre-Intervention			Post-Intervention		
	**Exams Performed**	**Patients Seen**	**Percent**	**Exams Performe d**	**Patients Seen**	**Percent**
Stiell 1994 - Use						
Ankle	544	657	83%	354	593	60%
Foot	204	657	31%	159	593	27%
Stiell 1995 - Use						
Overall	5207	6288	82.8%	3955	6489	60.9%

	Pre-Intervention			Post-Intervention		
	**Fractures**	**Patients Seen**	**Fractures per Patients Seen**	**Fractures**	**Patients Seen**	**Fractures per Patients Seen**
Stiell 1994 – Prevalence						
All – Sig.	103	657	16%	96	593	16%
Sig. Ankle	73	657	11%	81	593	14%
Sig. Foot	30	657	5%	20	593	3%
Avulsions	32	657	5%	20	593	3%
All Fractures	165	657	25%	116	593	20%
Stiell 1995 – Prevalence						
All – Sig.	1030	6288	16.4%	1082	6489	16.7%
Sig. Ankle	768	6288	12.2%	796	6489	12.3%
Sig. Foot	271	6288	4.3%	294	6489	4.5%
Avulsions	256	6288	4.1%	255	6489	3.9%
All Fractures	1286	6288	20.5%	1337	6489	20.6%
